# Achalasia in Children—Clinical Presentation, Diagnosis, Long-Term Treatment Outcomes, and Quality of Life

**DOI:** 10.3390/jcm10173917

**Published:** 2021-08-30

**Authors:** Dorota Jarzębicka, Piotr Czubkowski, Joanna Sieczkowska-Gołub, Jarosław Kierkuś, Adam Kowalski, Marek Stefanowicz, Grzegorz Oracz

**Affiliations:** 1 Department of Gastroenterology, Hepatology, Feeding Disorders and Pediatrics, The Children’s Memorial Health Institute, 04-730 Warsaw, Poland; d.jarzebicka@ipczd.pl (D.J.); joannasieczkowska@wp.pl (J.S.-G.); j.kierkus@ipczd.pl (J.K.); grzegorz@oracz.pl (G.O.); 2 Department of Pediatric Surgery and Organ Transplantation, The Children’s Memorial Health Institute, 04-730 Warsaw, Poland; a.kowalski@ipczd.pl (A.K.); m.stefanowicz@ipczd.pl (M.S.)

**Keywords:** myotomy, dysphagia, endoscopic dilatation, quality of life, Urbach scale, AAA syndrome, POEM

## Abstract

Background: In spite of the introduction of peroral endoscopic myotomy (POEM), Heller myotomy (HM) remains the mainstay of treatment and the role of pneumatic dilatation (PD) is being debated. The aim of this study was to present a single-center experience in the diagnostic approach and treatment of esophageal achalasia (EA), including the long-term assessment of the QoL. Methods: Data collection was based on the retrospective analysis of clinical notes and prospective interviews with patients and their parents. Results: The study group consisted of 60 patients with EA (F: 26, M: 34), with a median age of 12.0 (1–17) years at diagnosis. The time from the first symptoms until the diagnosis was 1.0 year (0.5–2.0) and the most common were: regurgitation (91.3%), dysphagia (84.8%), and chest pain (47.8%). The diagnostic approach showed a high sensitivity for barium X-ray follow through, esophageal manometry, and endoscopy. Overall, a long-term good outcome of HM was achieved in 27 out of 37 patients (73%) and it was negatively affected by the time between the first symptoms and the diagnosis. Out of the 16 patients who underwent PD before HM, a good outcome was achieved in 14 patients (87.5%), compared to 13 out of 21 patients (62%) who only underwent HM (*p* = 0.22). Concomitant fundoplication was routinely performed, and 18% required post-operative endoscopic dilatation. At the end of the 12.1 (0.7–26.6)-year follow up, most patients had a good QoL, which significantly corresponded with the treatment outcomes. Conclusions: Patients suspected of EA should undergo a thorough clinical evaluation including a manometry, a barium X-ray, and an endoscopy. HM is a safe and effective treatment for achalasia and the outcome is not worsened by a preceding endoscopic PD. In most patients, HM alleviates symptoms, although an impaired QoL is common in long-term follow ups.

## 1. Introduction

Esophageal achalasia (EA) is a rare primary esophageal motor disorder, caused by the degeneration or the lack of ganglion cells in the Auerbachian muscle layer in the lower esophagus. EA is characterized by an increased resting tension and an impaired swallow-induced relaxation of the lower esophageal sphincter (LES), which can cause ineffective esophageal peristalsis [1,2]. A recent epidemiological analysis of the pediatric population showed an incidence of 0.18/100,000 per year [3]. The occurrence of EA in very young children, especially <5 years of age, is extremely rare. It is also a component of AAA syndrome (alacrimia, achalasia, Addison disease), also called Allgrove syndrome, and is caused by a mutation in the *AAAS* gene located on the chromosome 12q13.8 [4]. The most common symptoms are dysphagia, retrosternal chest pain, regurgitation (or vomiting), and weight loss, but still, the diagnosis is often delayed in time [5]. These symptoms are included in the Eckardt Scoring Scale, which assesses the severity of symptoms in adults [6]. Treatment for EA aims to reduce the pressure of the LES so as to facilitate swallowing and improve the quality of life (QoL). Pharmacological treatment is ineffective, and there are also significant limitations of endoscopic botulinum toxin (BT) injections or pneumatic dilatation (PD). Surgical Heller myotomy (HM) was considered the gold standard due to its high effectiveness and safety [7]. Over the recent years, peroral endoscopic myotomy (POEM) was introduced as a minimally invasive technique, with a success rate similar to that of HM and with less adverse events [8]. There are reports in the literature suggesting that both techniques may be considered as first-line treatment options. Nevertheless, due to the limited access to POEM in children, reserved for a small number of specialized centers, and the short-term outcome measures, the real efficacy of this technique remains to be established [9]. So as to improve care for patients with EA, the International Society for Diseases of the Esophagus (ISDE) has established guidelines for the management of patients with esophageal achalasia, which outline the best practice in the treatment of EA [10].

The aim of this study was to present a single-center experience in the diagnostic approach and treatment of EA, including the long-term assessment of the QoL. Additionally, we described our experience with AAA syndrome patients.

## 2. Materials and Methods

### 2.1. Study Design

The study included 60 patients aged <18 years with diagnosed EA (a diagnosis based on clinical presentation, imaging, and functional tests), among them 9 patients with AAA syndrome, all treated in our hospital between 1996 and 2017. Patients with EA who did not undergo esophageal manometry but presented with the typical symptoms, a barium follow-through pattern, endoscopic findings, and the relief of symptoms after treatment, were included in the study. The protocol was approved by the Local Ethics Committee (234/KBE/2015). Data collection was based on the retrospective analysis of clinical notes and prospective interviews with the patients and parents who were asked to fill the questionnaires (in 2017), so as to assess the severity of symptoms: dysphagia (difficulty in swallowing and the feeling of a bite of food being stuck in the esophagus), regurgitation, heartburn, chest pain during swallowing, and coughing or choking during eating. The evaluation of tolerated foods included liquid, pulpy, plain, or ordinary diets with the necessity of drinking during the meals. The severity of symptoms was assessed according to their frequency per week and per month. Families were contacted over the phone and assisted in answering the questions. Based on the collected data, the long-term outcome of the treatment and the QoL were assessed. We did not include retrospective data on the long-term outcome of the treatment from clinical notes due to the quality of data, missing information, and the transition of patients to the other centers for adults.

### 2.2. Evaluation of Diagnostic Methods

All results of the investigations were re-evaluated with a senior radiologist or endoscopist and categorized according to a unified scoring system, as follows:

1. Barium X-ray follow through was evaluated by one radiologist on a radiological scale of EA according to Rezende et al. [11]: grade I—slow esophagus emptying, peristaltic disorders—tertiary waves or a lack of peristalsis; grade II—a slight enlargement of the esophagus, and more intense tertiary waves; grade III—a significant widening of the esophagus, with a narrowing of the lower segment—a characteristic image of the “bird’s beak”, violent convulsive muscle spasms or a complete lack of peristalsis of the esophagus; grade IV — the image as in stage III and a very large dilation of the esophagus with the change of its axis;
2. Gastroscopy results were evaluated according to the presence of the following: residual food in the esophagus, an enlargement of the esophagus, changes in the mucosa of the esophagus (resulting from long-residual food in the esophagus), stomach cardia—sometimes with a resistance passing the endoscope;
3. In the manometry, four basic features of EA were assessed: an increased resting pressure of the LES > 45 mmHg, a lack or incomplete LES relaxation in response to the incoming bite of food (LES > 8 mmHg), a lack of esophageal motility, and the positive pressure in the esophagus.

### 2.3. Treatment

#### 2.3.1. Endoscopic Pneumatic Dilatation

All procedures were performed by two senior endoscopists. In the majority, patients required a prompt intervention due to the high severity of symptoms, and PD was considered as a bridging therapy to HM. Under an X-ray control, the balloon was placed in the LES area and inflated until the narrowing was smoothed. The diameter of the balloon was selected depending on the patient’s age and severity of esophageal stenosis, usually between 15–40 mm. In this position, the balloon was held from 15–20 s to several minutes. During one session, the balloon was inflated 1 or 2 times. After the balloon removal, a routine endoscopic assessment was performed to check for a possible mucosa injury or an esophageal perforation.

#### 2.3.2. Heller Myotomy

HM [2,12] is the treatment of choice in our center. During laparotomy, longitudinal esophageal cardiomyotomy (5–6 cm) was performed, extending 1–3 cm into the gastric cardia, with concurrent fundoplication. All procedures were performed by experienced pediatric surgeons.

#### 2.3.3. Treatment Outcome

The treatment outcome was determined based on the occurrence of symptoms (dysphagia, regurgitation, heartburn, chest pain, and a cough or choking) after the treatment and the assessment of the QoL. To assess the QoL, patients were asked to answer the EA-oriented questions according to the Urbach scale [13]. The final result was calculated by summing up the points for each item and, thus, to yield a score between 10 and 31 (Table 1). The answers were valid only if there were no missing responses and were graded according to the rule: the lower the score, the better the QoL.

### 2.4. Statistical Analysis

Descriptive statistics of the demographic variables were presented using frequency, mean, or medians, with ranges or standard deviations, as appropriate. Comparisons of these variables were performed using the Fisher exact test for categorical variables and the Mann–Whitney test for continuous variables. A significant *p* value was <0.05.

## 3. Results

### 3.1. Demographics

The study group consisted of 60 patients with EA (F: 26, M: 34), with the median age of 12.0 years (a range of 1–17) at diagnosis. Co-morbidities occurred in 37 (61.7%) patients. The most common were GI (31.6%) and neurological disorders (15%). The median follow-up time was 12.1 years (0.7–26.6). Fourteen patients (23%) were lost to follow up. Overall, we were able to initiate contact with 46 patients (77%) with the median age of 22.9 years (6–41). Currently, 12 (20%) are below 18 years of age. In general, 29 patients (61.7%) are under gastroenterological care, 10 patients use proton pomp inhibitors (PPIs) and 1 patient uses prokinetics. Data are presented in Table 2.

### 3.2. Clinical Presentation at the Onset

The first symptoms occurred at the age of 9.4 years (0.1–17.5) and the median time until the diagnosis of EA was 1.0 year (0.5–2.0). The most common symptoms were: regurgitation (91.3%), dysphagia (84.8%), chest pain (47.8%), coughing or choking while eating (37%), and heartburn (21.7%). The severity of the main symptoms is presented in Table 2. Most of the patients (79.3%) tolerated a liquid or pulpy diet and only 20.6% tolerated an ordinary diet with or without the need for drinking during the meal. The BMI index was <10th percentile in 25 (41.6%) patients, <3rd percentile in 17 (28.3%) patients, and >90th in 4 (6.7%) patients. The mean BMI z-score at diagnosis was −1.7 ± 3.4. Weight loss before diagnosis was observed in 26% of cases.

### 3.3. Diagnostic Investigations

Overall, 40 patients (66.7%) underwent a full diagnostic evaluation, including a barium X-ray follow through, a gastroscopy, and an esophageal manometry. At least one typical barium X-ray feature was present in all patients (100%). The most common were the slow transition of contrast in 96.1%, esophageal dilation in 94.1%, and the “bird’s beak” sign in 82.4%. Esophageal manometry was performed in 41 patients and was consistent with EA in 95.1% of patients. Typically, LES pressure was >45mm in 24 patients (58.5%). Four patients did not undergo manometry due to the problematic passage of the catheter tip through the esophageal critical stricture. For another 15 patients, manometry was unavailable at the time of the clinical evaluation. Endoscopy showed abnormalities in 86.8% and the most common finding (75.5%) was of residual food in the esophagus (Table 3).

### 3.4. Treatment

**Figure 1:**
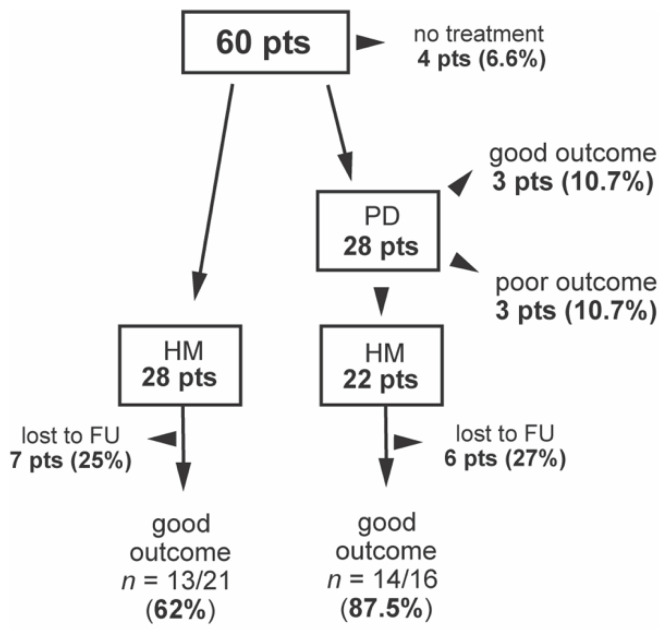
Outcomes of treatment. HM—Heller myotomy, NT—no treatment, PD—pneumatic dilatation, lost to FU—lost to follow up, pts—patients.

A total of 28 patients (46.7%) initially underwent HM and 28 (46.7%) primarily underwent endoscopic PD, and for 22, this was followed by HM (Figure 1). Overall, 50 patients underwent surgery (45 by laparotomy and 5 by laparoscopy), 98% with concomitant fundoplication. The median age at the operation time was 12 years (3–18). Four patients were not treated at all: two patients with AAA syndrome who did not report any GI symptoms and two patients who were lost to follow up (one after their transition to the adult center).

There were five complications after surgery: esophageal perforation in three patients (6%) and diverticulum in two (4%) patients. After HM, nine patients (18%) with fundoplication required endoscopic dilatation.

### 3.5. Long-Term Outcome and Prognostic Factors

Out of 56 patients who underwent treatment, 13 (23%) were lost to follow up and 43 (77%) patients were contacted at the mean age of 22.9 years (11 pts < 18 years of age) and 12 years (12, 0.7–26.6) after intervention. There were 37 patients after HM and 6 after PD without subsequent surgery.

According to the symptoms at the moment of contact with patients, a good outcome was defined as symptoms not occurring more often than once a week or four times per month, and a poor outcome was if at least one of the symptoms occurred for at least 5 days of the week.

Overall, a long-term good outcome of HM was achieved in 27 out of 37 patients (73%) but there were differences according to the initial treatment. Out of 16 patients who underwent PD before HM, a good outcome was achieved in 14 patients (87.5%), compared to 13 out of 21 patients (62%) who only underwent HM (Figure 1). Six patients underwent PD without HM, with a good outcome in three (50%) patients. Several variables were compared between patients with good and poor outcomes of HM. Although not significantly, the median time from the first symptoms to the initial treatment was longer in patients with a poor outcome (2.0 vs. 4.6 years, *p* = 0.11). Additionally, LES > 45 mmHg at diagnosis correlated with a poor outcome (*p* = 0.07). Other parameters were similar in both groups (Table 4).

### 3.6. Quality of Life

After receiving consent, the QoL questionnaires were obtained from 43 patients who answered the questions according to the Urbach scale [13]. The mean score was 16.4 and it significantly corresponded with good treatment outcomes (14.5 vs. 20.5, *p* > 0.0001). The most common symptoms reported by patients with a poor outcome were the need to drink while eating (100%), the limitation of certain foods that the patient was able to eat (80%), and pain while eating (50%). Half of the patients were not satisfied with their health in regard to achalasia, and 40% of patients reported a limitation of lifestyle because of EA (Table 3).

### 3.7. AAA Syndrome Patients

There were nine patients with AAA syndrome (six boys, three girls) with the median age of 5 (1–15) years at the time of diagnosis. The first symptoms were usually related to adrenal insufficiency, with the majority of the hypoglycemia occurring in infancy, while GI symptoms occurred at the median age of 11.0 years. Eight patients were contacted, and one was lost to follow up. Five patients were treated surgically, three of them with a good treatment outcome (60%). Two patients were not treated at all because of no GI symptoms. One patient was lost to follow up after their transition to adult care.

## 4. Discussion

In our study, we presented a single-center experience on pediatric EA. These data add significantly to the current evidence, which is sparse in regard to children and mostly comes from adult centers (10).

EA may have an insidious onset with a varying clinical presentation which can result in its delayed diagnosis and treatment. The most common symptoms are dysphagia, vomiting, chest pain, and malnutrition [14,15]. Some patients may present with refractory respiratory tract infections, as a result of aspiration to the respiratory system. The literature describes the occurrence of dysphagia in over 90% of patients, vomiting in 76–91%, chest pain in 17–95%, and heartburn in 27–42% [14,15,16]. In our cohort, only 20% tolerated a normal diet and almost 30% of patients presented with a BMI < 3rd percentile. The time from the first symptoms to the diagnosis is 2 years on average but may even be prolonged to 5 years [14,15]. If left untreated, EA may lead to the deterioration of the patient’s condition, malnutrition, and even to cachexia. In our study, we confirmed the high sensitivity of esophageal manometry, barium X-ray follow through, and endoscopy. Barium X-ray is often the first examination in the diagnostic approach with the effectiveness of 87–95%. However, in the initial phase of the disease, the result may be normal [17,18]. It also helps to exclude the causes of secondary achalasia, such as mediastinal tumors, and to monitor the patient’s condition after EA treatment [1,10]. Gastroscopy allows us to show any characteristic changes but also to initiate treatment. Typical findings comprise of residual food and mucosal changes due to chronic irritation. There is also the finding of a tightly closed LES which does not open with air insufflation [1]. The gold standard in EA diagnosis remains manometry, with an efficacy of over 90% [10,18]. High-resolution manometry allows us to distinguish achalasia types according to the Chicago classification [14,19].

Currently, effective endoscopic and surgical techniques are available, although there is no defined therapeutic algorithm for the pediatric population. According to the ISDE’s guidelines, treatment with BT is not recommended as the first-choice treatment in children [10]. The effectiveness of endoscopic PD is limited, and 30–75% of children require subsequent surgery due to recurrent symptoms [19,20,21,22]. Half of our patients underwent endoscopic PD as a first intervention, but the majority eventually required HM. A good outcome of HM was observed more frequently if preceded by PD, compared to HM alone. This observation was not statistically significant but allows us to consider PD as a bridging therapy to alleviate dysphagia before myotomy. This approach was previously reported as having a negative impact on HM outcomes [23,24] but is in line with the ISDE’s recommendations, as well as with the previous pediatric series which shows the efficacy of PD as a first-line approach [10,25,26].

The POEM technique is considered as a highly effective and safe method with an average success rate of 99.3%, compared to 77.9% of HM, and 44.9% for PD [9]. Nevertheless, due to the limited access, it has not replaced HM, especially in children where the evidence is sparse and limited to highly specialized centers. According to the ISDE’s guidelines, the effectiveness of POEM is comparable to that of surgeon treatment [10]. In our study, 90% of patients underwent classic HM, with a good long-term outcome and an improved QoL in the majority of the patients. Due to the period in which the study was conducted (1996–2017), laparoscopy gradually replaced laparotomy and remains the treatment of choice in our institution. Current evidence does not clearly state whether all children should undergo a concomitant anti-reflux procedure during HM. The main concern is the post-operative recurrence of dysphagia. Most centers perform anti-reflux procedures with a low rate of complications [27], although some series show no need for concomitant fundoplication [28,29,30]. In our experience, 18% of patients required subsequent endoscopic dilatation after fundoplication due to recurrent dysphagia. According to adult recommendations, partial fundoplication should be added to laparoscopic myotomy (10). One of the strengths of our study is the assessment of the long-term QoL performed by direct contact with patients and families. Most of the patients at the time of contact were over 18 years of age; therefore, we applied the adult Urbach scale. Because of the small proportion of patients under 18 years of age, it may be considered as a partial study limitation. The most common complaint was the need to drink while eating and these symptoms significantly affected daily life in 50% of patients.

In our study, we also presented the subgroup of patients with AAA syndrome which supplemented the current evidence. Our observations are consistent with previous studies where symptoms and treatment outcomes were similar to the rest of the patients [4,31,32]. The development of GI symptoms may be delayed in time, and adrenal insufficiency is usually diagnosed first. An early recognition of the syndrome is challenging due to the rarity of the condition and the high phenotypic heterogeneity [33].

Study limitations result mostly from the retrospective nature of data collection. Patients were often transitioned to local care, adults or were lost to follow up. Thus, the results of treatment are biased by the proportion of patients lost to follow up (30.2%). Due to the missing data, we were not able to perform an analysis of the early outcome of treatment, but we relied on cross-sectional long-term outcomes based on symptomatology and the QoL assessment. Due to the retrospective data, we could not use the Eckardt scale to quantify the severity of symptoms. An additional limitation was the inability to present the manometry results according to the Chicago classification.

## 5. Conclusions

In conclusion, patients suspected of EA should undergo a thorough clinical evaluation, including a manometry, a barium X-ray, and an endoscopy. In our experience, HM is a safe and effective treatment for achalasia and the outcome is not worsened by preceding endoscopic PD. There are no significant prognostic factors affecting the results of the treatment. However, worse outcomes may correlate with a delayed diagnosis. In most patients, HM alleviates symptoms, although an impaired QoL is common in long-term follow ups.

## Figures and Tables

**Table 1:** Quality of life assessment according to Urbach scale questionaries [13].

1. How much has achalasia limited the types of food you have been able to eat in the last month? (Please check one.)					
Not limited at all (I can eat and drink all the foods that I would like to). (1)		Somewhat limited (I can eat and drink most of the foods that I would like to). (2)		Moderately or severely limited (I can eat and drink very few of the foods that I would like to). (3)	
2. Raw hard fruits and vegetables: (please circle one.)					
Can swallow with no problem (1)		Can swallow, but with a little difficulty (2)		Can swallow with great difficulty or not at all (3)	
3. Rice: (Please circle one.)					
Can swallow with no problem (1)		Can swallow, but with a little difficulty (2)		Can swallow with great difficulty or not at all (3)	
4. Clear fluids (water, juice, coffee, tea): (please circle one.)					
Can swallow with no problem (1)		Can swallow, but with a little difficulty (2)		Can swallow with great difficulty or not at all (3)	
5. How often in the past month have you needed to drink water while eating to deal with food caught in your esophagus? (Please circle one.)					
Never/Rarely (1)		Sometimes (2)		Frequently/Every time I eat (3)	
6. How often have you experienced pain when eating during the past month? (Please circle one.)					
Never (1)	Rarely (2)	Sometimes (3)		Frequently/Every time I eat (4)	
7. During the past month, how much of a problem for you was heartburn (a burning pain behind the lower part of the chest)? (Please circle one.)					
No problem (1)	Mild problem (2)	Moderate problem (3)		Severe problem (4)	Very severe problem (5)
8. When you sit down to eat a meal, are you bothered by how long it takes you to finish eating? (Please check one.)					
No, I eat as quickly as I like. (1)			Yes, I am bothered by how long it takes me to eat. (2)		
9. Has having achalasia limited your lifestyle? (Please check one.)					
No, it is not at all limiting (My daily activities have not changed.) (1)			Yes, it has limited my lifestyle (It has affected some areas, and I can no longer participate in all the activities I want to do.) (2)		
10. How much do you agree with the following statement about how satisfied you are with your health in regard to achalasia? (Please circle the number that best describes your feelings.) I am satisfied with my health in regard to achalasia.					
Strongly agree (1)	Agree (2)	Neither agree or disagree (3)		Disagree (4)	Strongly disagree (5)

**Table 2:** Demographics, clinical presentation, and diagnostic tests.

Patient Data	Number/Mean (Median, Range)
Sex (male/female)	34/26
Type of EA	
Isolated	51 (85%)
AAA	9 (15%)
Age at first EA symptoms median/range (years)	9.4 (0.1–17.5)
Age at diagnosis median/range (years)	12.0 (1–17)
Time to EA diagnosis median/range (weeks)	1.0 (0.5–2.0)
Age at surgery (years)	11.7 (12, 3–18)
Age at the moment of follow-up contact (years)	22.9 (6–41)
Follow-up time (years)	12.1 (0.7–26.6)
Co-morbidities	37 (61.7%)
GI	19 (31.6%)
H. pylori infection	9 (47%)
Gastritis	3 (16%)
Duodennitis	2 (10%)
Celiac disease	2 (10%)
Pylorostenosis	2 (10%)
Gilbert syndrome	1 (5%)
Esophageal diverticula	1 (5%)
Neurological disorders	9 (15%)
Epilepsy	5 (55%)
Psychomotor retardation	4 (44%)
CMV infection with changes in EEG	1 (11%)
Muscular hypotonia	1 (11%)
Down syndrome	2 (3%)
Symptoms	*n* = 46
Dysphagia	39 (84.8%)
Regurgitation	42 (91.3%)
Retrosternal chest pain while eating	22 (47.8%)
Heartburn	10 (21.7%)
Coughing or choking while eating	17 (37%)
Tolerated diet	*n* = 58
Liquid	30 (51.7%)
Pulpy	16 (27.6%)
Ordinary	6 (10.3%)
Ordinary with the need for drinking while eating	6 (10.3%)
BMI	
Mean ±SD	16.3 ± 3.8
BMI z-score mean ± SD	−1.7 ± 3.4
<10th percentile	25 (41.6%)
<3rd percentile	17 (28.3%)
X-ray follow through	*n* = 51
EA features	51 (100%)
“bird’s beak” sign	42 (82.4%)
Esophageal dilatation	48 (94.1%)
Slow contrast transition	49 (96.1%)
Contrast retention in esophagus	38 (74.5%)
Esophageal peristalsis disorders	32 (62.7%)
Gastroscopy	*n* = 53
Any EA feature	46 (86.8%)
Residual food in the esophagus	40 (75.5%)
Esophageal enlargement	31 (58.5%)
Closed stomach cardia	39 (73.6%)
Esophageal mucosa lesions	15 (28.3%)
Manometry	*n* = 41
EA features	39 (95.1%)
LES > 45 mmHg	24 (58.5%)
Lack or incomplete LES relaxation	32 (78%)
Lack of esophageal motility	39 (95.1%)
Positive pressure in the esophagus	28 (68.3%)

EA—esophageal achalasia; AAA—alacrimia, achalasia, Addison disease; CMV—cytomegalovirus; EEG—electroencephalogram; BMI—body mas index; SD—standar deviation; LES—lower esophageal sphincter.

**Table 3:** Severity of symptoms at diagnosis.

	Regurgitation	Dysphagia	Chest Pain
Every Day	80.9%	64.70%	35.90%
Sporadic	10.4%	20.10%	11.90%
Absent	8.7%	15.20%	52.20%

**Table 4:** Comparison of clinical variables between patients with good and poor outcome of HM.

Variable	Good (*n* = 27)	Poor (*n* = 10)	95% CI	*p*-Value
Gender: female	13 (48%)	5 (50%)	0.63–1.49	0.99
AAA syndrome	3 (11%)	2 (20%)	0.30–1.30	0.59
Age at first GI symptoms				
years, median (range)	9.0 (0.1–15.5)	6.4 (0.7–14.7)	−5.7–1.7	0.35
Age at surgery				
years, median (range)	12.7 (3.0–18.7)	13.7 (5.2–17.2)	–2.9–3.6	0.80
Time from GI symptoms to initial treatment				
years, median (range)	2.0 (0.02–7.2)	4.6 (0.2–9.1)	–0.48–5.59	0.11
Manometry (*n* = 27)				
LES pressure >45 mmHg	12 (57%)	6 (100%)	0.43–0.99	0.07
Barium X-ray follow through (*n* = 33)				
Rezende III or IV grade	18 (75%)	5 (55%)	0.82–2.56	0.39
Endoscopy (*n* = 34)				
Mucosal changes	7 (26%)	3 (42%)	0.46–1.21	0.39
Residual food	17 (63%)	6 (85%)	0.57–1.23	0.38
BMI z-score at surgery				
<3 percentile	8 (29%)	3 (30%)	0.57–1.47	0.99
Treatment				
Endoscopic PD before HM	9 (33%)	1 (10%)	0.85–1.93	0.22

CI—confidence interval; AAA—alacrimia, achalasia, Addison disease; GI—gastrointestinal; LES—lower esophageal sphincter; BMI—body mas index; PD—pneumatic dilatation; HM – Heller myotomy.

## Data Availability

No new data were created or analyzed in this study. Data sharing is not applicable to this article.
